# Golden Ratio Gain Enhancement in Coherently Coupled Parametric Processes

**DOI:** 10.1038/s41598-018-30014-7

**Published:** 2018-08-02

**Authors:** Ottavia Jedrkiewicz, Alessandra Gatti, Enrico Brambilla, Martin Levenius, Gintaras Tamošauskas, Katia Gallo

**Affiliations:** 1 Istituto di Fotonica e Nanotecnologie del CNR, Udr Como, Via Valleggio 11, 22100 Como, Italy; 2 Dipartimento di Scienza e Alta Tecnologia, Università dell’Insubria, Via Valleggio 11, 22100 Como, Italy; 3 Department of Applied Physics, KTH – Royal Institute of Technology, Roslagstullsbacken 21, SE-10691 Stockholm, Sweden; 4 Laser Research Centre, Vilnius University, Saulėtekio 10, LT-10223 Vilnius, Lithuania

## Abstract

Nonlinear optical processes are an essential tool in modern optics, with a broad spectrum of applications, including signal processing, frequency conversion, spectroscopy and quantum optics. Ordinary parametric devices nevertheless still suffer from relatively low gains and wide spectral emission. Here we demonstrate a unique configuration for phase-matching multiple nonlinear processes in a monolithic 2D nonlinear photonic crystal, resulting in the coherent parametric emission of four signal and idler modes, featuring an exponential gain enhancement equal to the Golden Ratio. The results indicate a new route towards compact high-brightness and coherent sources for multi-photon generation, manipulation and entanglement, overcoming limitations of conventional parametric devices.

## Introduction

The Golden Ratio,$\,\phi =\frac{1+\surd 5}{2}$, is a sort of magic number, appearing in the most diverse fields of art^1,2^, biology^3,4^, mathematics^5–7^, physics and engineering^8–16^, often in connection with fractal growth and self-similarity. This work shows for the first time its spontaneous occurrence in a nonlinear optical process, in the form of a growth-rate enhancement of light generation. The relevant process is optical parametric generation (OPG)^17,18^, a paradigm for sources of classical and quantum-entangled light^19–24^.

OPG implies the spontaneous conversion of a photon from a high-energy pump laser (at frequency ${\omega }_{p}$) into a pair of lower-energy photons, at frequencies ${\omega }_{a}$ and ${\omega }_{b}$^17,18^. This occurs in a medium possessing a quadratic optical nonlinearity and can lead to an exponential build-up of the signal and idler fields, provided that photon energy (${\omega }_{p}={\omega }_{a}+{\omega }_{b}$) and momentum are preserved^25^. The latter condition is nowadays routinely enforced by quasi phase-matching (QPM) with nonlinear artificial structuring^26–28^.

**Figure 1:**
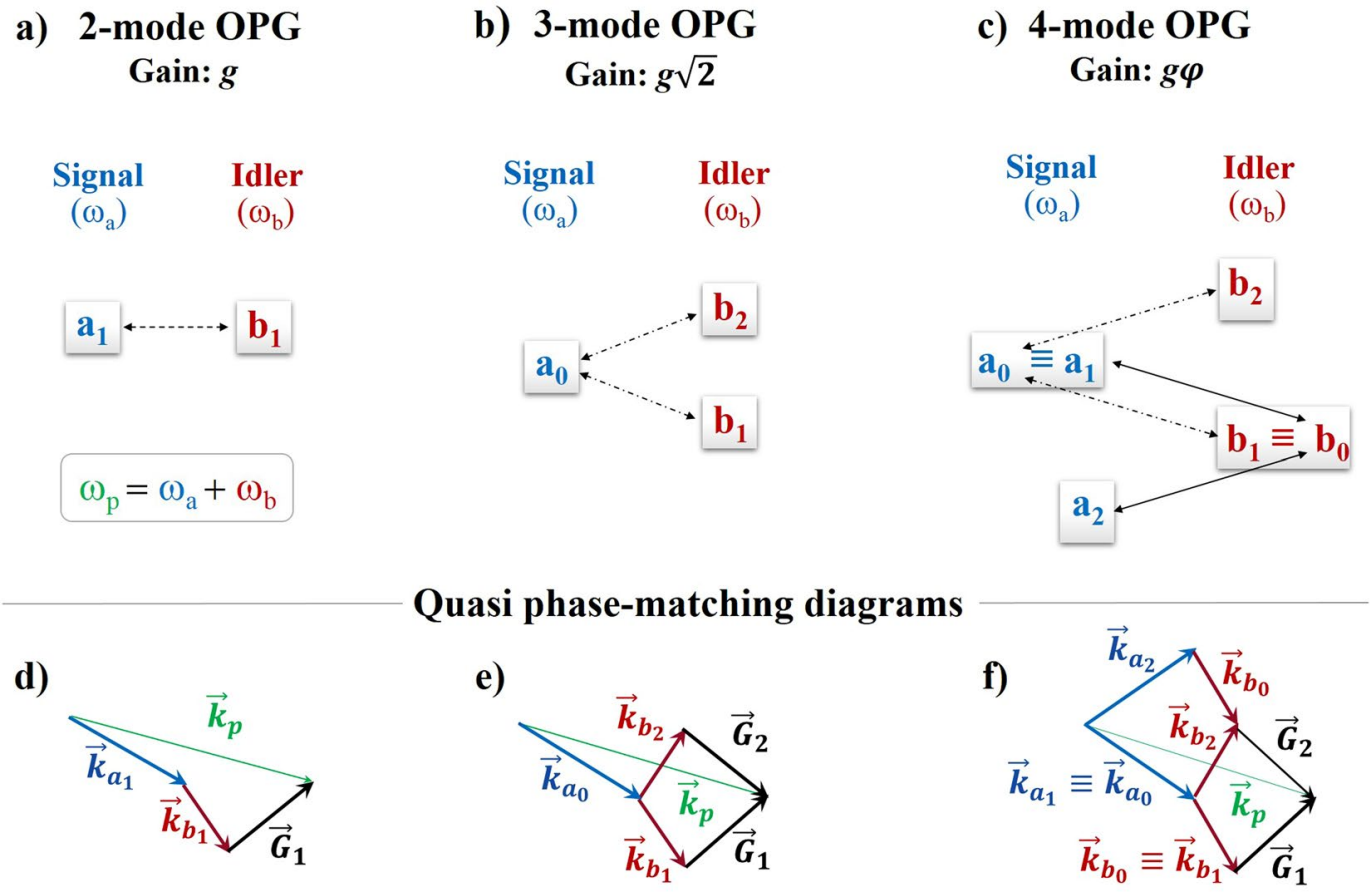
Multimodal optical parametric generation. Schematic interaction diagrams for signal (blue) and idler (red) modes. (**a)** Standard twin-beam (2-mode) OPG involving one signal (${a}_{1}$) and one idler (${b}_{1}$) mode. (**b)** 3-mode OPG involving a shared signal (${a}_{0}$) and two coupled idler modes (${b}_{1}$ and ${b}_{2}$). (**c)** 4-mode OPG involving both a shared signal (${a}_{0}$) and a shared idler (${b}_{0}$): the former is coupled to two idler modes (${b}_{2}$ and ${b}_{1}\equiv {b}_{0}$) and the latter to two signal modes (${a}_{2}$ and ${a}_{1}\equiv {a}_{0}$). QPM diagrams for: (**d)** 2-mode, (**e)** 3-mode and (**f)** 4-mode OPG.

Standard twin-beam (2-mode) OPG couples one signal and one idler mode, ${a}_{m}\,$and ${b}_{m}$, where the subscript *m* designates the mode pair, as illustrated in Fig. 1a (with *m* = 1). QPM among the wave-vectors of the pump, signal and idler, i.e. ${\overrightarrow{{\boldsymbol{k}}}}_{p},{\overrightarrow{{\boldsymbol{k}}}}_{{a}_{1}}$ and ${\overrightarrow{{\boldsymbol{k}}}}_{{b}_{1}}$, respectively, is then typically achieved through the momentum (${\overrightarrow{{\boldsymbol{G}}}}_{1}$) provided by a 1D nonlinear grating, so that: ${\overrightarrow{{\boldsymbol{k}}}}_{p}={\overrightarrow{{\boldsymbol{k}}}}_{{a}_{1}}+{\overrightarrow{{\boldsymbol{k}}}}_{{b}_{1}}+{\overrightarrow{{\boldsymbol{G}}}}_{1}$, as illustrated by the vectorial diagram of Fig. 1d.

More advanced interaction schemes may be afforded by nonlinear photonic crystals^27,28^, where a two-dimensional patterning of the quadratic nonlinearity provides multiple reciprocal lattice vectors, sustaining several concurrent QPM processes. Figure 1b illustrates the case of 3-mode OPG^29–32^, in which two idler modes, ${b}_{1}$ and ${b}_{2}$, are coherently coupled to a shared signal, ${a}_{0}$. The index *m* = 0 designates here the shared mode in the overall OPG output. In this case, as shown in Fig. 1e, QPM of two concurrent OPG processes is achieved via two reciprocal lattice vectors of the nonlinear photonic crystal (${\overrightarrow{{\boldsymbol{G}}}}_{1}\,$and ${\overrightarrow{{\boldsymbol{G}}}}_{2}$), satisfying the following conditions^29^:

$$\{\begin{array}{c}\,{\overrightarrow{{\boldsymbol{k}}}}_{p}={\overrightarrow{{\boldsymbol{k}}}}_{{a}_{0}}+{\overrightarrow{{\boldsymbol{k}}}}_{{b}_{1}}+{\overrightarrow{{\boldsymbol{G}}}}_{1}\\ \,{\overrightarrow{{\boldsymbol{k}}}}_{p}={\overrightarrow{{\boldsymbol{k}}}}_{{a}_{0}}+{\overrightarrow{{\boldsymbol{k}}}}_{{b}_{2}}+{\overrightarrow{{\boldsymbol{G}}}}_{2}\end{array}$$

Similarly, 3-mode OPG can also be sustained by a shared idler, ${b}_{0}$, coherently coupled to two signal modes, ${a}_{1}$ and ${a}_{2}$, quasi phase-matched via ${\overrightarrow{{\boldsymbol{G}}}}_{1}\,$and ${\overrightarrow{{\boldsymbol{G}}}}_{2}$, respectively^29–31^.

Here we use the QPM degrees of freedom of a 2D nonlinear photonic crystal to achieve OPG with four coupled output modes (two signals and two idlers), accessing a *super-resonant* regime, which may have some analogy with coherent effects observed in a different context^33^, whereby the parametric emission rates are enhanced as a result the coherent interplay of multiple parametric interactions. The waves in the nonlinear crystal interact according to the diagram of Fig. 1c, where a single pump excites three concurrent OPG processes, simultaneously generating three signal-idler mode pairs: (${a}_{0},\,{b}_{2})$, $({a}_{0},\,{b}_{0})$ and $({a}_{2},\,{b}_{0})$. This unique OPG configuration is underpinned by the following vectorial QPM conditions (Fig. 1f):

1 $$\{\begin{array}{c}{\overrightarrow{{\boldsymbol{k}}}}_{p}={\overrightarrow{{\boldsymbol{k}}}}_{{a}_{0}}+{\overrightarrow{{\boldsymbol{k}}}}_{{b}_{2}}+{\overrightarrow{{\boldsymbol{G}}}}_{2}\\ {\overrightarrow{{\boldsymbol{k}}}}_{p}={\overrightarrow{{\boldsymbol{k}}}}_{{a}_{0}}+{\overrightarrow{{\boldsymbol{k}}}}_{{b}_{0}}+{\overrightarrow{{\boldsymbol{G}}}}_{1}\\ {\overrightarrow{{\boldsymbol{k}}}}_{p}={\overrightarrow{{\boldsymbol{k}}}}_{{a}_{2}}+{\overrightarrow{{\boldsymbol{k}}}}_{{b}_{0}}+{\overrightarrow{{\boldsymbol{G}}}}_{2}\end{array}\,$$

where the subscript ‘0’ denotes the shared (signal and idler) modes, coupled via ${\overrightarrow{{\boldsymbol{G}}}}_{1}$, and the subscript ‘2’ denotes the remaining output modes, coupled via ${\overrightarrow{{\boldsymbol{G}}}}_{2}$, as depicted in Fig. 1f.

## Theoretical predictions

In the limit of perfect QPM, described by equations (1), plane-wave, monochromatic and undepleted pump, the evolution of the signal and idler modes in the crystal is approximately described by the following set of equations:

2 $$\{\begin{array}{l}\begin{array}{l}{\partial }_{z}{a}_{0}=\,g\,({b}_{0}^{\dagger }+{b}_{2}^{\dagger })\,\\ \begin{array}{l}{\partial }_{z}{a}_{2}=\,g\,{b}_{0}^{\dagger }\\ {\partial }_{z}{b}_{0}=\,g\,({a}_{0}^{\dagger }+{a}_{2}^{\dagger })\end{array}\end{array}\\ {\partial }_{z}{b}_{2}=\,g\,{a}_{0}^{\dagger }\end{array}\,$$

where *z* is the propagation variable and ${\partial }_{z}$ the *z-*derivative.$\,{a}_{m},\,\,{b}_{m}$ denote photon annihilation operators (or, equivalently, complex field amplitudes in a classical description)^29^ for the signal and idler modes at frequencies ${\omega }_{a}$ and ${\omega }_{b}$, respectively.

For standard OPG (Fig. 1a), in the same parametric limit under which equations (2) are derived, the mean photon-numbers in the high gain regime (*gz* ≫ 1) evolve in propagation as:$\,{N}_{{a}_{1}},{N}_{{b}_{1}}\sim {e}^{2gz}$, featuring an exponential growth factor equal to 2*g*^25^.

For the 3-mode OPG case (Fig. 1b), implemented with a shared signal (or, equivalently, with a shared idler), the mean photon-numbers evolve as: ${N}_{{a}_{0}},{N}_{{b}_{1}},\,{N}_{{b}_{2}}\sim \,{e}^{2\sqrt{2}gz}$, with an exponential gain enhancement of $\sqrt{2}$ with respect to standard OPG^32^.

The 4-mode parametric coupling of Fig. 1c, described by equations (2), instead, gives rise to an asymptotic exponential growth of the form ${N}_{{a}_{0}},{N}_{{a}_{2}},{N}_{{b}_{0}},\,{N}_{{b}_{2}}\sim {e}^{2\phi gz}$, where $\phi$ is the Golden Ratio (see also Methods). The appearance of this number can be traced back to the peculiar asymmetric form of the coupling, where the populations of modes $\,{a}_{0}$ and ${b}_{0}$ originate from two possible frequency down-conversion processes, while modes ${a}_{2}$ and ${b}_{2}$ are populated by a single OPG process (Fig. 1c).

## Experiments

**Figure 2:**
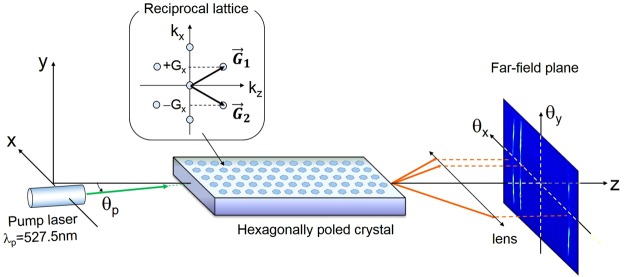
OPG interaction geometry in a hexagonally poled crystal. Sketch of the experimental configuration, with a pump beam propagating at a small angle (${{\boldsymbol{\theta }}}_{{\boldsymbol{p}}}$) to the symmetry axis of the nonlinear lattice (*z*). The axes *x* and *y* denote the transverse directions in real space. The inset highlights the two fundamental reciprocal lattice vectors (${\overrightarrow{{\boldsymbol{G}}}}_{1}$ and ${\overrightarrow{{\boldsymbol{G}}}}_{2}$) for the nonlinearity modulation in the *x-z* plane, used for QPM.

The 4-mode OPG concept was implemented in a hexagonally poled crystal, with a single pump beam at a fixed wavelength (${\lambda }_{p}$), which propagates at a small angle (${\theta }_{p}$) with respect to the symmetry axis of the nonlinear lattice (*z*), as sketched in Fig. 2.

The nonlinear medium was a nearly stoichiometric Z-cut periodically poled LiTaO_3_ crystal, doped with 1 mol% MgO (Oxide Corp)^34^. The pattern consisted of a hexagonal lattice of ferroelectric domains, with total inverted area ~33% and period Λ = 8.3 μm, fabricated by room temperature electric field poling^35^. The nonlinear chip was 2 cm-long, 4 mm-wide and 500 µm-thick along its X, Y and Z crystallographic directions, corresponding to the *z, x* and *y* axes, respectively, in the reference frame adopted in what follows. The crystal input and output facets were polished to optical grade, parallel to the (*x*, *y*) plane. The sample was mounted on a rotation stage and held at a constant temperature (*T* = 80 ± 0.1 °C) throughout the experiments.

The pump for the experiments was an 11 ps, 527.5 nm wavelength source, obtained from the second harmonic of a 1055 nm pulsed Nd:Glass laser, having a 10 Hz repetition rate. The input field was polarized along the *y*-axis (Z in the crystal frame) to make use of the largest nonlinear coefficient of LiTaO_3_, i.e. *d*_33_ = 16 pm/V^35^. The pump beam was shaped into an elliptical transverse profile and loosely focused along the vertical (*y*) direction. The beam size at the entrance of the crystal was 2 × 0.2 mm^2^. Twin beams in the near-infrared and telecom ranges (*λ*_*a*_ ~ 800 nm for the signal and *λ*_*b*_ ~ 1550 nm for the idler) were generated during the high-gain OPG process. During the experiment a complete characterization of the far-field signal radiation was performed, including a spectral and angular mapping of the OPG response in the near infrared for different pulse energies (10–100 μJ) and incidence angles (−2.5° to 2.5°) of the pump beam. The idler radiation was also monitored by means of an infrared camera (Xeva, Xenics) to verify the emission in the correct wavelength range, further confirming the expected OPG distributions (see Methods for details).

## Results and Discussion

**Figure 3:**
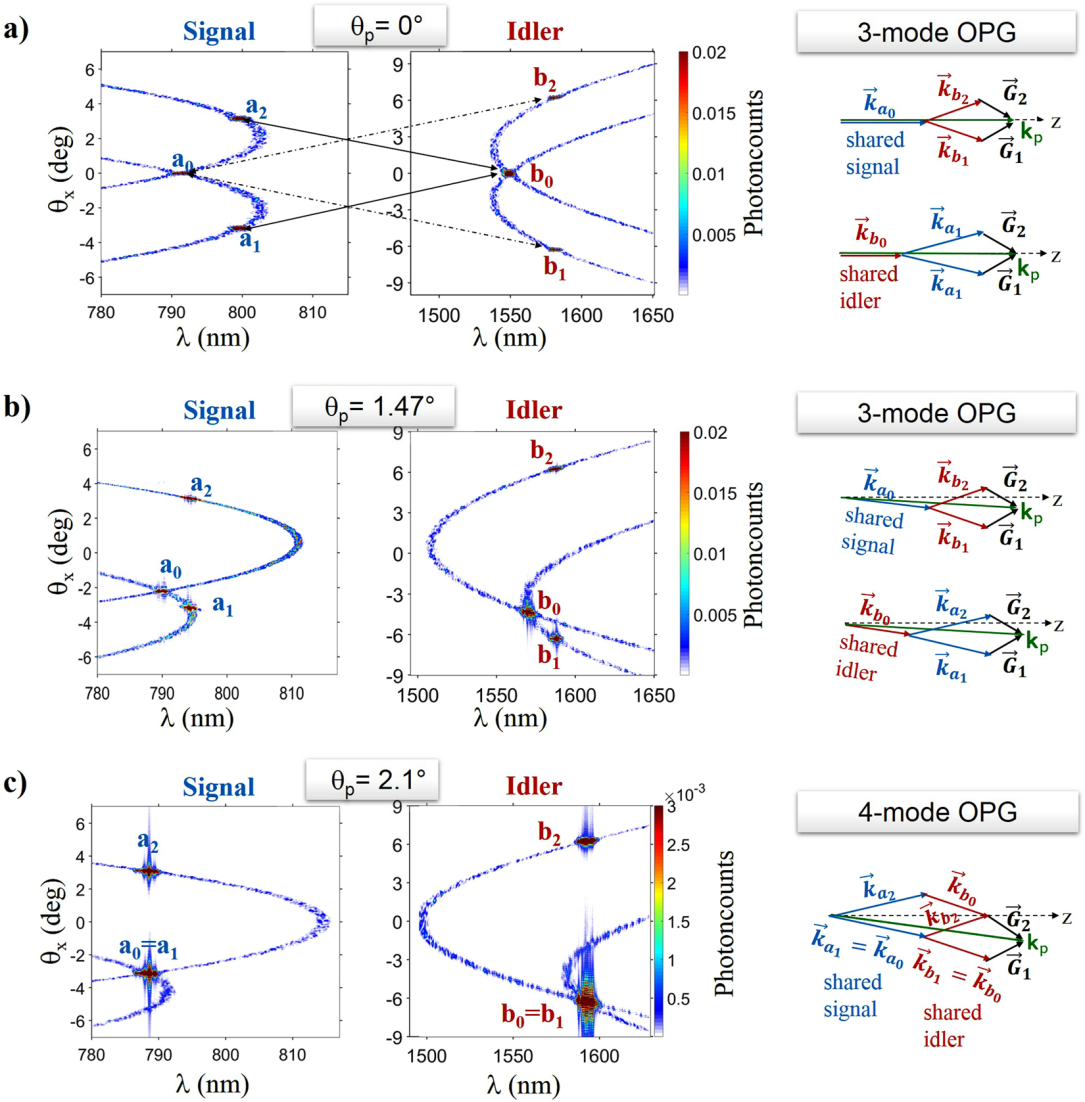
Theoretical predictions for multimodal OPG in hexagonally poled LiTaO_3_. Left and central columns: results of 3D+1 stochastic simulations, showing the single-shot photon-number distributions of the output signal and idler, respectively, as a function of wavelength and angle ${\theta }_{x}$, for ${\theta }_{y}={0}^{{\rm{o}}}$, with a pump at *λ*_p_ = 527.5 nm. The vertical scales have been truncated at 2% (**a,b**) and 0.3% (**c**) of the peak value, in order to show the standard 2-mode fluorescence, much weaker than the 3- and 4-mode hot-spots. (**a)** Symmetric (*θ*_p_ = 0^o^) and (**b)** asymmetric (*θ*_p_ = 1.47^o^) OPG, where the *hot spots* ${a}_{0},\,{b}_{1},\,{b}_{2}$ and ${b}_{0},{a}_{1},{a}_{2}$ realize the 3-mode coupling conditions with a shared signal and a shared idler, respectively. (**c)** Asymmetric OPG for *θ*_p_ = 2.1^o^, corresponding to the super-resonance condition. The two triplets of hot spots coalesce into 4 coupled modes, realizing the interaction scheme of Fig. 1c. The diagrams on the rightmost column illustrate the QPM conditions at the work points for 3-mode and 4-mode OPG.

QPM in the nonlinear photonic crystal was achieved through the fundamental reciprocal lattice vectors ${\overrightarrow{{\boldsymbol{G}}}}_{1}\,$and ${\overrightarrow{{\boldsymbol{G}}}}_{2}$ (inset of Fig. 2), for which: $|{\overrightarrow{{\boldsymbol{G}}}}_{1}|=|{\overrightarrow{{\boldsymbol{G}}}}_{2}|=4\pi /({\rm{\Lambda }}\sqrt{3})$. Each of them supports, for a given value of ${\theta }_{p}$, infinite sets of QPM solutions, defined in terms of the wavelength ($\lambda$) and transverse propagation angles $({\theta }_{x},{\theta }_{y}$) of the signal and idler modes. In the 3D ($\lambda ,{\theta }_{x},{\theta }_{y})$ parameter space, the signal (idler) solutions lie on two surfaces, corresponding to QPM via ${\overrightarrow{{\boldsymbol{G}}}}_{1}$ or ${\overrightarrow{{\boldsymbol{G}}}}_{2}$, respectively. Their intersection with the plane of the lattice (${\theta }_{y}=0$) yields two ($\lambda ,{\theta }_{x}$) branches were OPG emission is concentrated, as illustrated by Fig. 3.

Figure 3a and b, show the numerical results of a realistic modelling of the experiments (see also Methods) for 3-mode OPG with symmetric (${\theta }_{p}=0$) and asymmetric pumping, respectively. Both cases make apparent the existence of OPG *hot-spots* at the intersections of the two QPM branches, where output intensities are significantly enhanced. This is ascribed to 3-mode OPG, consistently with previous findings^29–31^. The labels highlight the coupling of a shared signal (${a}_{0}$) to two symmetric idler modes (${b}_{1}$, ${b}_{2}$) and the dual case, i.e. a shared idler (${b}_{0}$) coupled to two signal modes (${a}_{1}$, ${a}_{2}$), according to the previously introduced notation. The diagrams on the R.H.S. illustrate the corresponding momentum conservation laws.

As the pump is tilted away from the symmetry axis (${\theta }_{p}=1.47$°, Fig. 3b), the shared signal (idler) mode gets closer to one of the coupled signal (idler) modes, i.e.: ${a}_{0}\to {a}_{1}$ (${b}_{0}\to {b}_{1}$) until, for a given pump incidence angle (${\theta }_{p}=2.1$°, Fig. 3c), a peculiar *super-resonant* condition is met, where the *x*-components of all the optical wave-vectors (pump, signal and idler) are individually matched to the *x*-component of either ${\overrightarrow{{\boldsymbol{G}}}}_{1}$ or ${\overrightarrow{{\boldsymbol{G}}}}_{2}$. The interacting optical fields then experience the same spatial transverse modulation as the crystal lattice, resulting in constructive interference of the parametric emission in the specific directions satisfying equations (1). The coherence of the pump in the (*x, z*) plane is also expected to play a role, in analogy with other superradiance emission phenomena^33^. Specifically, the longitudinal and transverse coherence lengths of the pump, here coinciding with its extensions in the *z* (~1.5 mm) and *x* (2 mm) directions, must be much larger than the poling period (Λ ~ 8.3 *μ*m) of the pattern. Accordingly, the expected nonlinear interference effect is clearly observed even if the pump pulse length is sub-optimal with respect to temporal walk-off between the pump and idler pulses (duly accounted for in the simulations), occurring over a distance ${L}_{p-i}\sim 14$ mm (shorter than the overall propagation length: *L* = 20 mm).

When the super-resonant condition is met, the two triplets of OPG modes, originally uncoupled (Fig. 3b), coalesce into 4 coupled modes (Fig. 3c). This enables the interaction scenario of Fig. 1c, whereby 2 modes of the signal (${a}_{0}$ and ${a}_{2}$) and 2 of the idler (${b}_{0}$ and ${b}_{2}$), pairwise frequency-degenerate, realize the 4-mode coupling of equations (2).

**Figure 4:**
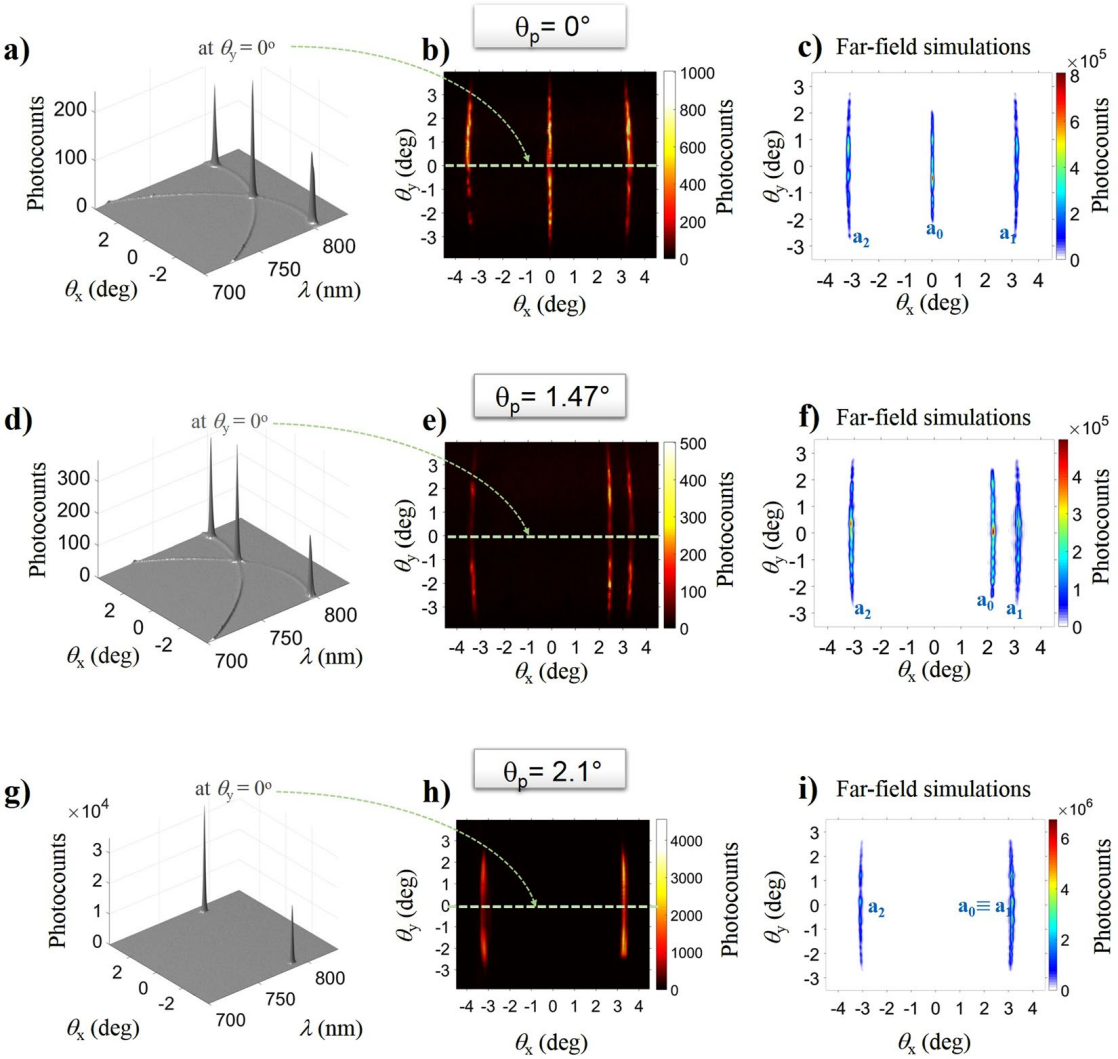
Control of the OPG coupling by angular tuning. Spectral-angular $({\theta }_{x},\,\lambda )$ distributions of the output signal radiation measured for ${\theta }_{y}={0}^{{\rm{o}}}$, i.e. in the lattice plane (leftmost column**)** and far-field $({\theta }_{x},\,{\theta }_{y})$ signal distributions from measurements (central column) and simulations (rightmost column). Results obtained for a fixed pump energy, E_p _ = 30 μJ, and three different pump incidence angles: (**a–c)** ${\theta }_{p}={0}^{0}$, (**d–f)** ${\theta }_{p}={1.47}^{0}$, and (**g–i)** ${\theta }_{p}={2.1}^{0}$. By tilting the pump incidence angle from the symmetric configuration to the superresonance angle, the three hot-spots seen in the $({\theta }_{x},\,\lambda )$-maps (**a,d**) coalesce into two (**g**). The effect is accompanied by a marked enhancement in OPG brightness and by the merging of the three lines seen in the far field (**b,c**,**e,f**) into two (**h,i**). The experimental data were obtained by recording the signal radiation over 20 laser shots. The numerical simulations show single-shot results.

To access in the experiments the *super-resonant* regime, the pump propagation direction was adjusted by rotating the crystal. For each pump incidence angle, a full 3D characterization of the signal fluorescence was performed at the output, by measuring the intensity angular distributions in the far-field and their spectral-angular distributions in the *x* and *y* directions. The key experimental results are illustrated by Fig. 4(a–i), corresponding to ${\theta }_{p}={0}^{{\rm{o}}},\,\,{1.47}^{{\rm{o}}}$ and ${2.1}^{{\rm{o}}}$, respectively, i.e. the three configurations analyzed in Fig. 3. The hot spots of 3 and 4-mode OPG were order of magnitudes brighter than the surrounding 2-mode OPG background (barely visible in the plots of Fig. 4a,d and g). For 3-mode OPG, three nearly straight lines were observed in the far-field plane (Fig. 4b and e): a central line featuring all the hot-spots associated with a shared signal (*a*_0_), and two outer lines featuring those of the signals coupled to a shared idler (*a*_1_, *a*_2_), in full agreement with simulations (Fig. 4c and f). The wavelength of the radiation emitted along the hot-spot lines varied over a broad range (Fig. s3, Supplementary Information).

As the crystal was rotated away from the symmetric pumping condition (Fig. 4b,c), the central (shared signal) line approached one of the outer lines (Fig. 4e,f). Their merging at the *super-resonance* condition (Fig. 4h,i) was accompanied by a sudden and dramatic increase of the hot-spot intensity (Fig. 4g). In these conditions, modes ${a}_{0}$ and ${a}_{1}$ become fully degenerate (in both wavelength and angle) as confirmed by the measurements of Fig. 4g.

**Figure 5:**
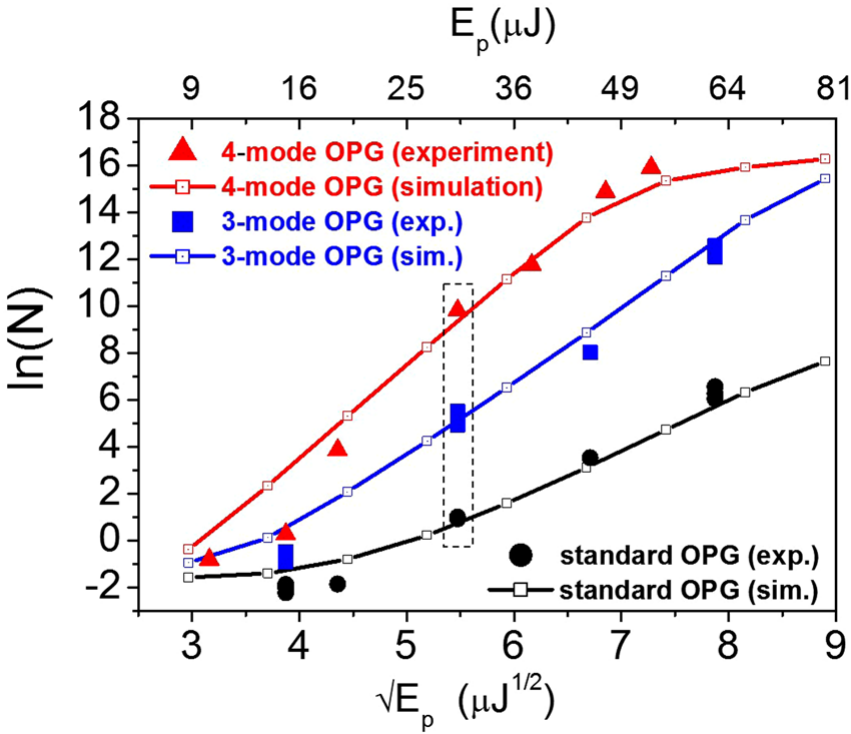
Gain enhancement of coherently coupled OPG processes. Signal photon numbers at the output, plotted in logarithmic scale versus the square-root of the pump energy (E_p_). The markers show experimental data, while the open symbols connected by solid lines are the result of numerical simulations. The dashed rectangle highlights the working points corresponding to Fig. 4 (E_p_ = 30 μJ). Red triangles and blue squares show measurements at the hot-spots for 4-mode and 3-mode OPG, respectively. For comparison, the results for standard 2-mode OPG are also shown as black circles (error bars are smaller than the size of the symbols, see also Supplementary Information).

To confirm the predictions for the Golden Ratio gain enhancement, the evolution of the output signal intensities in 2-, 3- and 4- mode OPG was systematically investigated as a function of the pump energy in the experiments (Fig. 5). Based on a simplified analytical model [equations (2) and Methods] and on the proportionality of the parameter $g$ therein to the amplitude of the pump field^25,32^, an exponential growth of the output photon number with the square root of the input pump energy (${E}_{p})\,\,$is expected, i.e.: $N\propto {e}^{\gamma \sigma \sqrt{{E}_{p}}}$, where $\sigma \,\,$is a constant, while the pre-factor $\gamma$ takes the value of $1,\,\sqrt{2}\,\,$and $\phi =1.618\ldots$ for the case of 2-, 3- and 4-mode OPG, respectively. Accordingly, the OPG response is analyzed by plotting the values of $N$ in logarithmic scale as a function of $\sqrt{{E}_{p}}$. Figure 5 shows the experimental data for 2-, 3- and 4-mode OPG as: black circles, blue squares and red triangles, respectively. Open symbols connected by solid lines show the results of numerical simulations, based on a realistic modelling of the experiments (Methods).

Three distinct linear trends in the evolution of $\mathrm{ln}\,N$ are apparent in the 15–50 $\mu J$ energy range, with a marked increase in the slopes when moving from 2-, to 3-, and finally to 4- mode OPG. Linear fits on the experimental data yield gain enhancement factors: ${\gamma }^{(exp)}$ = 1.51 ± 0.38 and $\gamma$^(exp)^ = 2.07 ± 0.71 for 3-mode and 4-mode OPG, respectively, which compare well with numerical simulations, yielding: $\gamma$^(sim)^ = 1.47 ± 0.17 and $\gamma$^(sim)^ = 1.83 ± 0.17 in the two cases (details of the data analysis and fit procedure provided in Supplementary Information).

Overall the experimental results summarized by Fig. 5 agree, within the errors, with analytical predictions based on equations (2), even though the experimental conditions satisfy only very roughly the approximations implied by the analytical model. This is a further indicator of the robustness of the predicted physics. It is also noteworthy that the experimental data consistently deviate from the analytical predictions in the same direction as the results of the numerical simulations.

## Conclusion

In summary, we revealed the possibility of a unique four-mode coupling in optical parametric generation, resulting in a gain enhancement equal to the Golden Ratio, which stems from the peculiar symmetries of the nonlinear coupling. We realized it in a monolithic format by exploiting the quasi-phase-matching degrees of freedom of a hexagonally poled crystal. Experiments performed in the high-gain regime provided evidence for enhanced OPG emission with localized intensity hot-spots, in very good agreement with theoretical predictions. The results provide a new paradigm for tailoring gain and coherent emission in optical parametric amplifiers, oscillators and entangled photon sources, by engineering the coherent coupling of multiphoton processes. Further generalizations to even higher number of modes may be envisaged through the extra degrees of freedom afforded by nonlinear quasi-crystals, higher dimensionality lattices and excitation with structured pump beams.

## Methods

### Experiments

The emission from the crystal around the signal wavelength was first spatially characterized in the far field by means of a *f* = 50 mm focal length lens, placed at a distance *f* from the output face of the crystal, and the far field images were recorded by a high efficiency, 16 bit, charged-coupled-device (CCD) camera (Andor) placed in the lens focal plane (Supplementary Information Fig. s1). A razor edge filter with 99% transmission for wavelengths >530 nm was used to cut the pump. Neutral density filters were used to control the intensity level onto the CCD chip. Single shot and multiple shot images were recorded by controlling the exposure time of the CCD camera. The spectrally resolved angular spectrum of the signal radiation was then recorded by means of an imaging spectrometer (Lot Oriel) with a 150 μm-wide slit. This spectrometer, constituted by a grating placed in the center of a telescopic system made of two spherical mirrors, allows the reconstruction of the angular spectrum of the injected far-field radiation along the slit direction, and of the wavelength spectrum along the orthogonal direction (diffraction plane of the grating). The result is a 2D image recorded by a CCD camera that gives an angular and wavelength mapping of the OPG radiation^36^.

The signal emission was characterized both in the *θ*_x_ = 0 plane and in the *θ*_y_ = 0 plane (Supplementary Information Fig. s3). For the characterization of the emission in the horizontal plane, the far-field signal beam underwent a 90° rotation by means of two-beam steering mirrors and was then focused by the *f* = 50 mm lens onto the vertical slit of the imaging spectrometer. The spectra were recorded at the very output of the spectrometer (imaging plane of the slit plane), by the CCD camera (Supplementary Information Fig. s2).

### Theory and simulations

The OPG process is modelled in terms of coupled propagation equations for the three (pump, signal and idler) interacting field operators, described as wave-packets centered around their carrier frequencies. The periodic nonlinear modulation in the (*x, z*)-plane is described by keeping only the leading order terms in the Fourier expansion of the nonlinear susceptibility:$\,d(x,z)\approx {d}_{01}\,{e}^{i{G}_{z}z}({e}^{i{G}_{x}x}+\,{e}^{-i{G}_{x}x})$, where ${\overrightarrow{{\boldsymbol{G}}}}_{1,2}=\,{G}_{z}{\overrightarrow{{\boldsymbol{e}}}}_{z}\pm \,{G}_{x}{\overrightarrow{{\boldsymbol{e}}}}_{x}$ are the two fundamental vectors of the reciprocal lattice (Fig. 2)^27^. For the hexagonally poled crystal used in the experiments: ${G}_{z}=\frac{2\pi }{{\rm{\Lambda }}}$, ${G}_{x}=\frac{2\pi }{{\rm{\Lambda }}\sqrt{3}}$, and ${d}_{01}=0.29\,{d}_{33}$^30^. Propagation equations are written in the Fourier domain spanned by the 3D vector ${\boldsymbol{w}}=({k}_{x},{k}_{y},\,{\rm{\Omega }})$, where ${k}_{x},\,{k}_{y}$ are the transverse components of the field wave-vectors and ${\rm{\Omega }}$ is the frequency shift from the carrier frequencies^37,38^. They account for pump depletion, spatial and temporal walk-off, diffraction and dispersion at any order, and take the following form:

3 $$\begin{array}{c}{\partial }_{z}{A}_{s}({{\boldsymbol{w}}}_{s},z)=\chi \int {d}^{3}{{\boldsymbol{w}}}_{p}\,{A}_{p}({{\boldsymbol{w}}}_{p},z)[{A}_{i}^{\dagger }({{\boldsymbol{w}}}_{p}-{{\boldsymbol{w}}}_{s}-{{\bf{G}}}_{{\boldsymbol{x}}},z){e}^{-i{{\mathfrak{D}}}_{1}({{\boldsymbol{w}}}_{s},{{\boldsymbol{w}}}_{p})z}\\ \,\,\,\,\,+\,{A}_{i}^{\dagger }({{\boldsymbol{w}}}_{p}-{{\boldsymbol{w}}}_{s}+{{\bf{G}}}_{{\boldsymbol{x}}},z){e}^{-i{{\mathfrak{D}}}_{2}({{\boldsymbol{w}}}_{s},{{\boldsymbol{w}}}_{p})z}]\end{array}$$

4 $$\begin{array}{c}{\partial }_{z}{A}_{i}({{\boldsymbol{w}}}_{i},z)=\chi \int {d}^{3}{{\boldsymbol{w}}}_{p}\,{A}_{p}({{\boldsymbol{w}}}_{p},z)[{A}_{s}^{\dagger }({{\boldsymbol{w}}}_{p}-{{\boldsymbol{w}}}_{i}-{{\bf{G}}}_{{\boldsymbol{x}}},z){e}^{-i{{\mathfrak{D}}}_{1}({{\boldsymbol{w}}}_{p}-{{\boldsymbol{w}}}_{i}-{{\bf{G}}}_{x},{{\boldsymbol{w}}}_{p})z}\,\\ \,\,\,\,\,+\,\,{A}_{s}^{\dagger }({{\boldsymbol{w}}}_{p}-{{\boldsymbol{w}}}_{i}+{{\bf{G}}}_{{\boldsymbol{x}}},z){e}^{-i{{\mathfrak{D}}}_{2}({{\boldsymbol{w}}}_{p}-{{\boldsymbol{w}}}_{i}+{{\bf{G}}}_{{\boldsymbol{x}}},{{\boldsymbol{w}}}_{p})z}]\,\end{array}$$

5 $$\begin{array}{c}{\partial }_{z}{A}_{p}({{\boldsymbol{w}}}_{p},z)=-\,\chi \int {d}^{3}{{\boldsymbol{w}}}_{s}\,{A}_{s}({{\boldsymbol{w}}}_{s},z)[{A}_{i}^{\dagger }({{\boldsymbol{w}}}_{p}-{{\boldsymbol{w}}}_{s}-{{\bf{G}}}_{{\boldsymbol{x}}},z){e}^{i{\mathfrak{D}}{}_{1}({{\boldsymbol{w}}}_{s},{{\boldsymbol{w}}}_{p})z}\\ \,\,\,\,\,\,+\,{A}_{i}^{\dagger }({{\boldsymbol{w}}}_{p}-{{\boldsymbol{w}}}_{s}+{{\bf{G}}}_{{\boldsymbol{x}}},z){e}^{i{{\mathfrak{D}}}_{2}({{\boldsymbol{w}}}_{s},{{\boldsymbol{w}}}_{p})z}]\end{array}$$

where ${{\bf{G}}}_{{\boldsymbol{x}}}$ is a short-hand notation for the vector $({G}_{x},\,0,0)$ in ***w***-space, and ${A}_{j}({\boldsymbol{w}},z)$ are the positive frequency parts of the field operators for the signal $(j=s)$, idler $(j=i)\,\,$and pump $(j=p)$ beams, with dimensions such that $\langle {{A}_{j}}^{\dagger }{A}_{j}\rangle$ is a number of photons per unit frequency and wavevector-squared. The first and second term at the R.H.S. of equations (3–5) describe all the possible OPG processes mediated by the lattice vectors ${\overrightarrow{{\boldsymbol{G}}}}_{1}\,\,$and ${\overrightarrow{{\boldsymbol{G}}}}_{2}$, respectively. Their phase-matching functions ${{\mathfrak{D}}}_{1,2}({{\boldsymbol{w}}}_{s},{{\boldsymbol{w}}}_{p})={k}_{sz}({{\boldsymbol{w}}}_{s})+{k}_{iz}({{\boldsymbol{w}}}_{p}-{{\boldsymbol{w}}}_{s}\mp {{\bf{G}}}_{{\bf{x}}})-{k}_{pz}({{\boldsymbol{w}}}_{p})+{G}_{z}$ account for the efficiency by which each of these processes occurs, where ${k}_{jz}({\boldsymbol{w}})=\sqrt{{k}_{j}^{2}({\rm{\Omega }})-({k}_{x}^{2}+{k}_{y}^{2})}$ is the *z*-component of the wave-vector of the mode, with wavenumber ${k}_{j}({\rm{\Omega }})=\frac{{\omega }_{j}+{\rm{\Omega }}}{{\boldsymbol{c}}}n({{\rm{\omega }}}_{{\rm{j}}}+{\rm{\Omega }})$, *n* being the extraordinary refractive index of the nonlinear crystal^35^.

Numerical simulations were performed in the framework of the Wigner representation, where field operators are replaced by *c*-number fields^39^. The input signal and idler fields, initially in the vacuum state, are simulated stochastically with Gaussian white noise, while the injected pump field is a high-intensity coherent pulse. The equations are integrated with a pseudo-spectral (split-step) method^38^. The dimensions of the numerical grid used for the simulations (512 × 256 × 512 points along *x, y* and the temporal axis, respectively) were carefully chosen to accommodate the spectral and angular bandwidths of the pump (with Gaussian spatial and temporal profiles) as well as the generated signal and idler distributions, under realistic modelling conditions.

### Analytical model

Analytical results can be derived in the parametric limit, where the pump is approximated by a classical monochromatic plane-wave, undepleted by the OPG process.

We assume that the plane-wave pump propagates in the $(x,z)$-plane at a small angle ${\theta }_{p}={q}_{p}/{k}_{p}$ with the *z*-axis. As will be detailed in a further theoretical publication, the shared signal modes are then characterized by the same *x*-component of the wave-vector as the pump, ${k}_{x}={q}_{p}.$ In these conditions, when the pump is not tilted to super-resonance (${q}_{p}\ne \pm \,{{G}}_{x}$), the 3-mode OPG condition is realized. With a shared signal and for perfect phase-matching $({{\mathfrak{D}}}_{1}={{\mathfrak{D}}}_{2}=0)$, the configuration is described by the following set of equations:

6 $$\{\begin{array}{c}{\partial }_{z}{a}_{0}=g[{b}_{1}^{\dagger }+{b}_{2}^{\dagger }]\\ {\partial }_{z}{b}_{1}=g{a}_{0}^{\dagger }\\ {\partial }_{z}{b}_{2}=g{a}_{0}^{\dagger }\,\end{array}$$

where the coupling strength is $\,g=\chi {\alpha }_{p}=\frac{\sigma }{2L}\sqrt{{E}_{p}}$, ${\alpha }_{p}$ being the classical pump amplitude and $L$ the crystal length. These equations are analogous to those first derived and analyzed in a classical framework in ref.^32^. When applied to our specific lattice configuration, the mean photon numbers at the crystal output ($z=L$) are given by:

7 $$\,{N}_{{a}_{0}}(L)\sim {N}_{{b}_{1,2}}(L)\propto {\sinh }^{2}(\sqrt{2}gL)\simeq \frac{1}{4}{e}^{2\sqrt{2}gL}\,$$

where the last line holds in the high-gain regime $gL\gg 1$. The above expression is to be compared with the standard behavior of 2-mode OPG, where $\,N\propto {\sinh }^{2}(gL)\simeq \frac{1}{4}\,{e}^{2gL}$, Analogous results apply to the dual case of 3-mode OPG with a shared idler, with the substitutions: ${a}_{0}\to {b}_{0},\,{b}_{1}\to {a}_{1}$ and ${b}_{2}\to {a}_{2}$.

When the pump is tilted to super-resonance, i.e. for ${q}_{p}=\pm \,{{G}}_{x}$, two sets of 3-modes coalesce into a single set of 4 coupled modes, evolving in the sample according to the model described by equations (2) and equally valid for classical field amplitudes, and 4-mode OPG can be realized. Eigenvalues and eigenvectors of this 4-mode dynamical coupling can be found with some algebraic manipulations. In particular, for perfect phase matching the eigenvalues are $\pm g\phi$ and $\pm g/\phi$, where *φ* is the *Golden Ratio*. Remarkably, $g\phi$ and $g/\phi$ correspond to two independent parametric processes, one showing an enhancement of the growth rate of light $g\,\to \,g\times 1.618\,\mathrm{..}$ and the other a decrease $g\,\to g\times \mathrm{0.618...}$ with respect to standard 2-mode OPG. At high parametric gains the first process clearly dominates. Accordingly, in this regime the mean photon numbers exhibit the following asymptotic behaviors:

8 $${N}_{{a}_{0}}(L)={N}_{{b}_{0}}(L)\propto \frac{{\phi }^{2}}{1+{\phi }^{2}}{\sinh }^{2}(g\phi L)\,\simeq \frac{{\phi }^{2}}{4(1+{\phi }^{2})}{e}^{2\phi gL}$$

9 $${N}_{{a}_{2}}(L)={N}_{{b}_{2}}(L)\propto \frac{1}{1+{\phi }^{2}}{\sinh }^{2}(g\phi L)\,\simeq \frac{1}{4(1+{\phi }^{2})}{e}^{2\phi gL}$$

Therefore, when the chain of stimulated processes becomes long enough, the intensity of the hot-spots exhibits a power-law increase with respect to the surrounding 2-mode fluorescence ${N}_{{\rm{4}}-{\rm{mode}}}=\,{({N}_{{\rm{2}}-{\rm{mode}}})}^{\phi }$. These analytical predictions agree quite well with the experimental findings shown in Fig. 5, confirming the robustness of the predicted phenomena, despite the deviation of the experimental conditions from the approximations of the model, in particular due to the onset of temporal walk-off effects in this relatively long crystal.

### Data availability

Supplementary information is available in the online version of the paper.

## Electronic supplementary material

Supplementary Information
