# Supplementary material for: Golden Ratio Gain Enhancement in Coherently Coupled Parametric Processes
Source: Sci Rep. 2018 Aug 2;8:11616. doi: 10.1038/s41598-018-30014-7 (PMC6072770; doi:10.1038/s41598-018-30014-7)
Supplement: Supplementary file 1 — Supplementary Information [file 41598_2018_30014_MOESM1_ESM.docx]

**Supplementary Information**

**“Golden Ratio Gain Enhancement in Coherently Coupled Parametric Processes”**

Ottavia Jedrkiewicz,^1^ Alessandra Gatti,^1,2^ Enrico Brambilla,^2^ Martin Levenius,^3^ Gintaras Tamošauskas,^4^ and Katia Gallo^3*^^[[1]](#footnote-1)^

**Characterization of the OPG signal radiation**

**1. Far-field spatial measurements**

The far-field measurement technique consists in recording in the imaging plane of a lens the angular distribution of the emitted radiation from the output of the crystal. Multiple shots or single shot images could be recorded by the CCD (Andor), in the latter case given the laser repetition rate (10 Hz) by setting a detection window of 100 ms. The CCD background was acquired before each measurement and automatically subtracted by the CCD software during each acquisition.


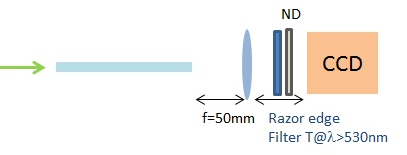


pump

NPC

**Figure s1**: Schematics of the detection apparatus for the signal far-field radiation based on the use of a CCD camera.

**2. Spectrally resolved angular measurements**

The acquisition of spectrally resolved angular measurements was performed by means of an imaging spectrometer coupled to the CCD camera described above. The detection efficiency of the whole system comprising also of an imaging interferometer was lower than in the case of the far-field measurements. In this case in order to highlight the presence of the fluorescence branches of the 2-mode OPG process, a temporal integration was usually performed.

**Figure s2**: Schematics of the spectral-angular detection of the signal radiation based on the use of an imaging spectrometer and a CCD camera.

NPC

grating

Parabolic mirrors

CCD

f=50mm

**3. Spectral-angular 3D** $\left( \boldsymbol{\theta}_{\boldsymbol{x}}\mathbf{,}\boldsymbol{\theta}_{\boldsymbol{y}}\mathbf{,}\boldsymbol{\lambda} \right)$**maps**

Far-field spatial measurements and spectrally-resolved angular measurements were combined to yield full 3D spectral angular mappings for each experimental working point, i.e. at a given incidence angle (*θ*_p_) and energy (*E*_p_) of the pump beam impinging on the nonlinear crystal. This is illustrated by Fig. s3 below, for the case of *θ*_p_ = 0 and *E*_p_ = 30 μJ.

**
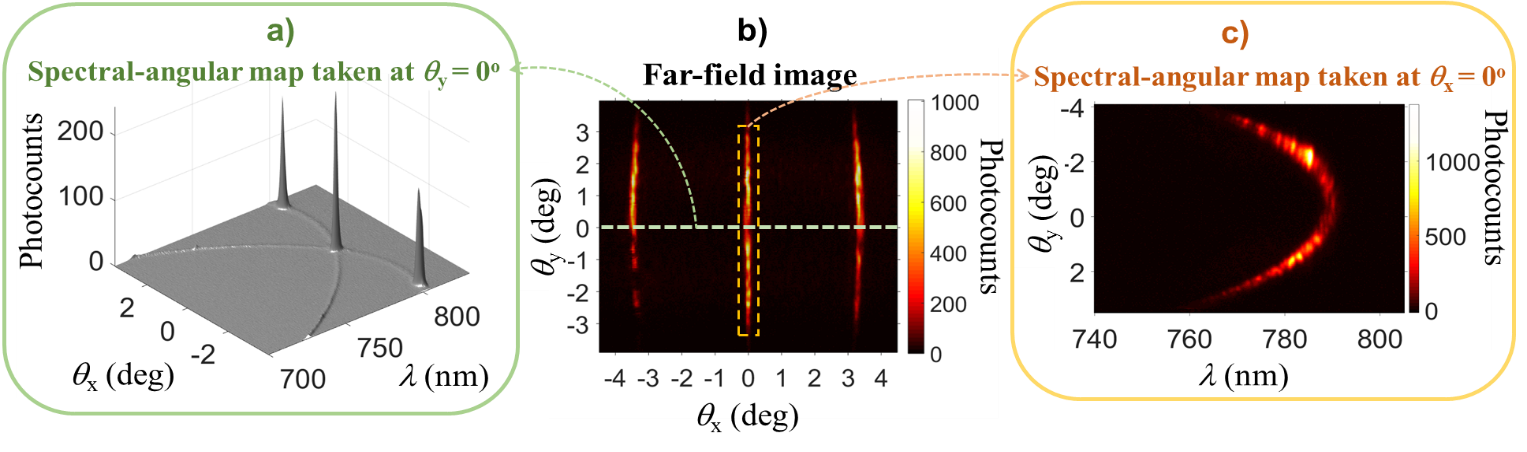
**First, far field signal output measurements were performed with the setup of Fig. s1 (results in Fig. s3b). Then, the spectral contents of the horizontal line corresponding to emission in the plane of the 2D nonlinear lattice ($\theta_{y}$ = 0, Fig. s3a) and of the vertical hot-spot lines observed at given values of $\theta_{x}$ (e.g. $\theta_{x}=0$ in Fig. s3c) were separately retrieved with the imaging spectrometer of Fig. s2.

**Figure s3**: Experimental mapping of the $\left( \theta_{x}, \theta_{y},\lambda\right)$OPG signal response retrieved from combined sequential measurements of far-field $\left( \theta_{x}, \theta_{y} \right)$, $\left( \theta_{x}, \lambda\right)$ and $\left( \theta_{y}, \lambda\right)$ intensity distributions, performed with the systems of Fig. s1 and s2. Experimental data retrieved for $\theta_{p}=0$, *E*_p_ = 30 μJ.

**Estimation of the gain enhancement γ (data analysis in Fig. 5)**

We illustrate here in some detail how the exponential enhancement factor γ for 3- and 4- mode OPG has been inferred from the experimental data set and compared with the results of the numerical simulations as shown in Fig.5. Each point in the graph of Fig.5a corresponds to a single acquisition of the signal spectral-angular distribution integrated over multiple pump shots. Figure s4 shows two typical images recorded from the CCD at the output of the imaging spectrometer, from which we estimated the photocounts associated with standard 2-mode OPG and 3-mode OPG (panel a), and to 4-mode OPG (panel b), respectively.

**
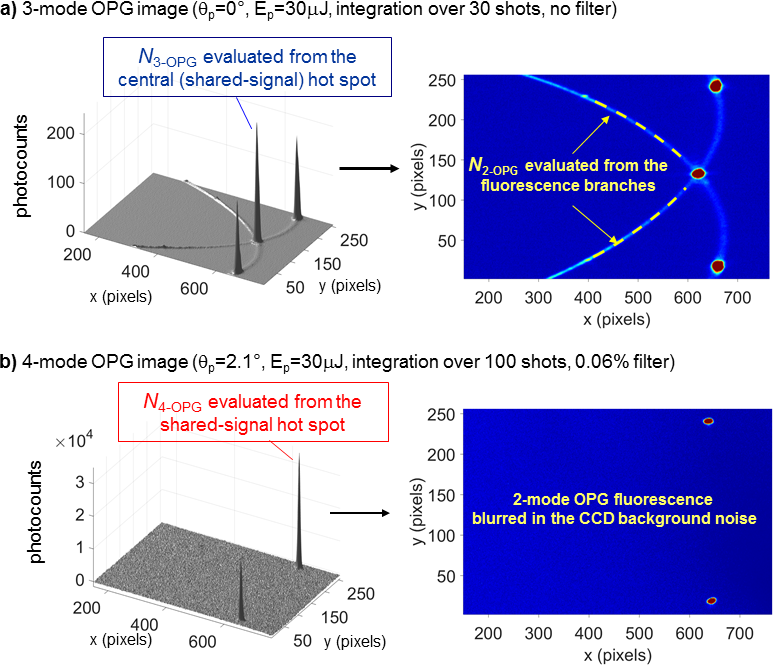
**

**Figure s4**: Typical CCD images retrieved from a single acquisition of the signal spectral-angular distribution for a) 3-mode OPG and b) 4-mode OPG. In case a) the two fluorescence branches appear under the three hot spots; the plot on the right also displays the pixels over which *N*_2-OPG_ has been estimated (dashed lines). In case b) only the two hot spots from 4-OPG are visible.

Notice that the photocounts in each image have been rescaled in order to take into account the corresponding CCD integration time and the transmission of the used density filters. Different filters with transmission in the range 10^-4^ – 10^-1^ were used to maintain the hot spots photocounts within the CCD dynamical range. Their choice depended both on the pump energy and on the integration times which varied from 1 up to 20 seconds (corresponding to 10-200 pump pulses).

For estimating the exponential growth rate associated with 3- and 4- mode OPG processes, we decided to consider the photocount peak value on the more intense hot spot corresponding to the shared-signal mode a_0_, (indicated with *N*_3-OPG_ and *N*_4-OPG_ respectively in Fig.s4). We verified that the choice of the other hot spot coupled to the shared-idler mode (mode a_2_ in Fig.3) led to similar results.

**
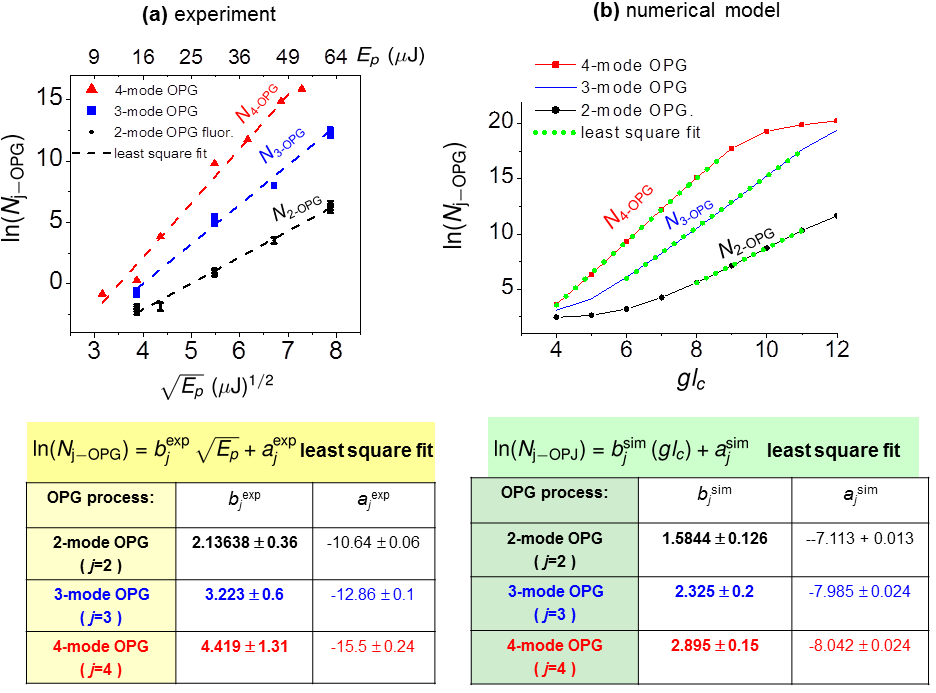
**

**Figure s5**: Result of the linear least square fit for the estimation of the gain enhancement γ for 3- and 4-mode OPG obtained from a) the experimental data b) the numerical model.

Photocounts from standard fluorescence were less straightforward to assess, since they are generally several orders of magnitude lower than those at the hot spots. In practice, standard fluorescence could be distinguished from the CCD background noise only in the case of 3-mode OPG operation. In the example shown in Fig.s4(a), the fluorescence photocount (*N*_2-OPG_) was obtained by averaging the pixel photocounts over ~360 pixels located on the two phase-matching branches (see dashed lines). This same procedure was applied to each acquisition and required a careful localization of the phase-matching branches on the CCD images. The standard errors estimated from those averages are indicated by error bars on the black symbols in Fig. s5(a) (in Fig.5a the size of the symbols is larger than the error bars).

Panel (a) in Fig.s5 summarizes the result of 18 acquisitions taken within a range of pump energies between 10 μJ and 64 μJ (same data set as in Fig.5a). The table in panel (a) reports the three slopes *b*_j_ obtained from a least square linear fit of ln(*N_j_*_-OPG_) vs $\sqrt{E_{p}}$for the three different OPG processes (i.e. for *j*=2,3,4). For comparison, we performed a similar linear fit for ln(*N_j_*_-OPG_) vs *gl_c_* from the results of the numerical simulations shown in panel (b). In the numerical model the gain parameter is defined as *g*=χ*α*_p_, where *α*_p_ is the peak value of the amplitude of the Gaussian pump pulse at the crystal input face: $A_{p}\left( x,y,t \right)=\alpha_{p}e^{-\frac{x^{2}}{\sigma_{x}^{2}}}$ $e^{-\frac{y^{2}}{\sigma_{y}^{2}}}$ $e^{-\frac{t^{2}}{\tau_{p}^{2}}}.$ Notice that *g* defined in this way coincides with the coupling parameter appearing in the analytical model in Eqs.(1) and (5) only in the limit of a plane-wave monochromatic pump. In the simulations, the dimensionless parameter *gl_c_* varied in the interval (4-12) (*l_c_* = 2 cm being the crystal length), while the pump waists and duration were taken at the fixed values *σ_y_* = 200 μm, *σ_x_* = 600 μm, and τ_p_ = 10 ps. The linear fits for estimating the exponential rate from the numerical curves have been done within regions where ln(*N*) grows almost linearly with *gl_c_* (dotted green lines in panel b). Following this guideline, we had to exclude large gain values in the case of 4-mode OPG (red line), since the effect of pump depletion becomes particularly evident for *gl_c_* > 8 (notice that pump depletion in 3-mode OPG conditions takes place at higher pump energies).

The experimental values of the gain enhancement γ^(exp)^ for 3- and 4-mode OPG reported in table of Fig.5 have obtained from the corresponding slope ratios, $b_{3}^{\exp}$/$b_{2}^{\exp}$=1.51 + 0.38 and $b_{4}^{\exp}$/$b_{2}^{\exp}$=2.07 + 0.71 respectively. Similarly, the values of γ^(sim)^ have been obtained from the ratios $b_{3}^{\mathrm{sim}}$/$b_{2}^{\mathrm{sim}}$=1.47 + 0.17 and $b_{4}^{\mathrm{sim}}$/$b_{2}^{\mathrm{sim}}$=1.83 + 0.17 inferred from the numerical simulations.

It is interesting to point out that the slope for 2-mode OPG obtained from the numerical simulation, $b_{2}^{\mathrm{sim}}$=1.58 ± 0.126, is below 2, the value predicted by the analytical model for *gl_c_* >> 1 (see Methods). This should not come as unexpected, however, since the simplified analytical model presented in Methods holds only in the ideal limit of a plane-wave monochromatic pump field. In the pulsed regime considered in the experiment and in the numerical model, the temporal walk-off of the signal and idler photons from the 11 ps pump pulse is relevant and reduces the effective OPG gain by about 20% according to the simulations. For example, we estimated that the idler photons have an average temporal walk-off with respect to the pump pulse $l_{c}/{2v}_{gp}-l_{c}/2v_{gi}=7.9$ ps (*v_gp_* and *v_gi_* denote the pump and idler group velocities at the reference frequencies). It is important to stress, however, that this reduction of the conversion efficiency affects the three different OPG processes by about the same amount. As demonstrated both experimentally and by the numerical model, the observed gain enhancements for 3- and 4-mode OPG are slightly greater than the predicted value of the analytical model ($\surd2$ and φ ≈1.618 respectively), thus appearing as a robust phenomenon.

We note that the numerical curves shown in panel (b) of Fig.s5 have been superimposed to the experimental data in Fig5a through two linear transformations which do not affect the ratio between their slopes, i.e. the enhancement factors γ : **i)** the *gl_c_*→ $\sqrt{E_{p}}$ mapping for the horizontal axis and **ii)** the rescaling of the numerical values of *N*_j-OPG_ by a common factor that accounts for the detection efficiency of the imaging spectrometer. The first transformation can in principle be performed through a theoretical estimation of the relation between *gl_c_* and $\sqrt{E_{p}}$ holding for the pump pulse used in the experiment. However, due to some uncertainty in the experimental parameters and the walk-off effect described above, we preferred an empirical approach and estimated the ratio *gl_c_*/$\sqrt{E_{p}}=\sigma/2$ needed for transformation **i)** by comparing the slopes of ln(*N*_2-OPG_) (i.e. for standard fluorescence) obtained in the simulations and in the experiment. In this way we obtained the value σ/2=$b_{2}^{\exp}$/$b_{2}^{\mathrm{sim}}$=1.35μJ^-1/2^. Transformation **ii)** is also empirically implemented by matching the values of ln(*N*_2-OPG_) and produces a common rigid downward translation of the three curves of about 4 units. Noticeably, this rigid translation produces an excellent matching between the numerical and experimental results also for 3- and 4-mode OPG.

1. Corresponding author: [gallo@kth.se](mailto:gallo@kth.se) [↑](#footnote-ref-1)
